# Engineering CD4+ T Cells to Enhance Cancer Immunity

**DOI:** 10.3390/cells9071721

**Published:** 2020-07-18

**Authors:** Francesca Sillito, Angelika Holler, Hans J. Stauss

**Affiliations:** 1 Division of Infection and Immunity, Institute of Immunity and Transplantation, University College London, Royal Free Hospital, London NW3 2PF, UK; 2 Cancer Institute, Royal Free Hospital, University College London, London NW3 2PF, UK; a.holler@ucl.ac.uk

**Keywords:** T cell receptor (TCR), T helper cell (Th), major histocompatibility complex (MHC), mechanistic target of Rapamycin 1 (mTORC1), programmed death receptor 1 (PD-1), interferon-gamma (IFN-γ)

## Abstract

This review presents key advances in combining T cell receptor (TCR) gene transfer to redirect T-cell specificity with gene engineering in order to enhance cancer-protective immune function. We discuss how emerging insights might be applied to CD4+ T cells. Although much attention has been paid to the role of CD8+ cytotoxic T cells in tumour protection, we provide convincing evidence that CD4+ helper T cells play a critical role in cancer immune responses in animal models and also in patients. We demonstrate that genetic engineering technologies provide exciting opportunities to extend the specificity range of CD4+ T cells from MHC class-II-presented epitopes to include peptides presented by MHC class I molecules. Functional enhancement of tumour immunity can improve the sensitivity of T cells to cancer antigens, promote survival in a hostile tumour microenvironment, boost cancer-protective effector mechanisms and enable the formation of T-cell memory. Engineered cancer-specific CD4+ T cells may contribute to protective immunity by a direct pathway involving cancer cell killing, and by an indirect pathway that boosts the function, persistence and memory formation of CD8+ T cells.

## 1. Introduction

Adoptive therapy with genetically engineered T cells allows for precision targeting of tumour antigens to treat a wide range of malignancies. Gene transfer techniques, commonly involving gamma retroviral or lentiviral vectors, have been developed to successfully transfer TCR genes into primary T cells and redirect their specificity towards cancer antigens [1,2].

More recently, zinc finger nuclease-based techniques have been employed to remove endogenous TCRs and improve the pairing and expression of the introduced TCR chains [3]. Clustered regularly interspaced short palindromic repeats (CRISPR)–Caspase 9 (Cas9) allows for precise genome editing using the protein Cas9, which binds with a guide RNA to create a molecular entity which can bind and cut DNA [4]. CRISPR-based engineering techniques have enabled the insertion of introduced TCR genes into the endogenous TCR locus in human T cells [5].

The TCR α and β chains form heterodimers that assemble with the CD3 γ, δ, ε and ζ chains and with the CD4 or CD8 coreceptors in helper and cytotoxic T cells, respectively. While the TCR–CD3 complex contains 10 immune-tyrosine activation motifs (ITAMs) that are important for efficient signal transduction and T-cell activation, most chimeric antigen receptor CAR constructs have only three ITAMs [6]. TCR-mediated T-cell activation depends on binding to peptides presented by MHC molecules, and the binding of the CD4 and CD8 coreceptors to MHC class II and class I molecules, respectively. Although TCR and coreceptor binding to peptide/MHC provides an essential first signal, it is not sufficient for full T-cell activation. A second costimulatory signal, frequently provided by the binding of CD28 to CD80 and CD86, enables T-cell activation and prevents the induction of anergy that is observed when T cells receive TCR signals in the absence of costimulation [7,8]. In addition to the TCR Signal 1 and the costimulation Signal 2, there is a further Signal 3 required for optimal T-cell activation and memory formation. Signal 3 is provided by soluble cytokines such as IL-2, IL-4, IL-7, IL-15 and IL-21, which can reduce apoptosis of activated T cells, promoting clonal expansion and memory formation [9].

T cells transduced with TCRs specific for tumour-associated antigens have demonstrated anticancer activity in clinical trials [10,11,12]. The most common cancer antigens that have been targeted in TCR gene therapy trials are New York ESOphageal squamous cell carcinoma 1 (NY-ESO-1), Melanoma Antigen Recognized by T cells (MART-1) and Wilms Tumour antigen 1 (WT-1) [13]. However, therapy with TCR-engineered T cells currently lags behind the use of T cells engineered to express chimeric antigen receptors (CARs), which have been remarkably effective in the treatment of CD19-expressing haematological malignancies [14]. This success, together with the fact that CAR recognition does not require a specific HLA genotype of patients, has resulted in substantial investment into clinical trials with CAR-engineered T cells. Although TCRs have the disadvantage of HLA restriction, which limits the number of patients that can be treated with the same TCR, they have the advantage of recognizing intracellular antigens that cannot be recognized by CARs. Unlike CARs, TCRs are also effective in recognizing intracellular mutated neoantigens, providing an opportunity to direct T cells against truly cancer-specific antigens that are absent in normal tissues.

## 2. Role of CD4+ T Cells in Cancer Immunity

To date, investigations of the role of T cells in cancer immunity have largely focused on CD8+ T cells. This is related to the observation that cancer cells usually express the major histocompatibility complex (MHC) class I molecules required for recognition by CD8+ T cells, but not MHC class II, which are required for antigen recognition by CD4+ T cells. In addition, many cancer-associated peptide epitopes recognized by CD8+ T cells have been identified, while the knowledge of ‘helper’ epitopes recognized by CD4+ T cells is relatively sparse. Finally, there has been an assumption that the ‘killing function’ of CD8+ T cells is more important in protection against cancer than the ‘helper function’ of CD4+ T cells.

However, there is a substantial amount of data indicating a key role of CD4+ T cells in cancer immunity. Studies in murine models have shown that effective CD8+ T cell responses against MHC class-II-negative tumours required the ‘helper function’ of CD4+ T cells [15]. More recently, it has been shown that CD4+ helper T cells can inhibit the expression of inhibitory receptors in CD8+ T cells [16], and that helper T cells are essential for the formation of functional CD8+ memory T cells [17,18,19]. In addition to providing essential ‘help’ for functional CD8+ T-cell responses, CD4+ T cells can also display killing activity and, when adoptively transferred in murine models, provide effective cancer immunity [20]. A recent analysis of antigen-specific immunity in cancer patients revealed that CD4+ T cells efficiently recognized mutated neoantigens in melanoma [21]. Furthermore, data from recent clinical trials suggest an important helper function of these neo-antigen-specific CD4+ T cells. The immune monitoring of patients who received neoantigen vaccines showed that the induction of antigen-specific CD4+ T-cell responses correlated with the stimulation of poly-functional CD8+ T-cell responses [22,23].

Together, the evidence above indicates that CD4+ T cells play a key role in cancer immunity, which provides a strong rationale to explore whether genetic engineering can be employed to optimize their contribution to effective tumour protection.

## 3. Stimulation of ‘Helper Function’ by Major Histocompatibility Complex (MHC) Class-II-Negative Tumours

The transfer of MHC class-I-restricted TCRs into CD4+ T cells provides an opportunity to stimulate helper T cells in tumours that lack the expression of MHC class II that is required for stimulation of ‘conventional’ helper T cells. However, in the absence of CD8 coreceptors, the peptide concentration required for stimulation of CD4+ T cells expressing a class-I-restricted TCR is approximately 10-fold higher than the concentration required for stimulation of CD8+ T cells expressing the same TCR [24]. The sensitivity of CD4+ T cells can be improved by transduction with high-affinity TCRs [25,26], or by the cotransfer of genes encoding TCR and CD8 [27,28]. Importantly, when CD4+ T cells transduced with class-I-restricted TCRs plus CD8 are stimulated with cells presenting the TCR-recognised MHC class-I-presented peptide antigen, they retain a cytokine production profile that is characteristic of CD4+ helper T cells and distinct from that of CD8+ cytotoxic T cells [24]. Thus, TCR plus CD8 engineering provides an opportunity to generate helper T cells that can efficiently respond to cancer antigens presented by MHC class-I-positive tumour cells.

## 4. Increasing T-Cell Response by Cytokine Engineering

Interleukin-12 (IL-12) is a potent proinflammatory cytokine that can enhance innate immunity and also boost responses by CD4+ and CD8+ T cells [29,30]. Due to the systemic toxicities associated with IL-12 administration [31,32,33], there has been some focus on developing safe IL-12 delivery systems that allow for controlled expression of IL-12 by engineered T cells. This can be achieved using Tet-on promoter systems responsive to doxycycline, allowing for tight control of IL-12 secretion. This controlled IL-12 secretion in T cells transduced with a melanoma-specific TCR was shown to be associated with an increase in the number of tumour-infiltrating lymphocytes and an improved protection from melanoma progression in a murine model [34]. The relative potency of IL-12 to boost cancer immunity when delivered by TCR-engineered CD4+ or CD8+ has not yet been explored.

T cells engineered to express IL-18 under the control of the nuclear factor of activated T-cell (NFAT) promoter were tested in a preclinical model. Kunert et al. demonstrated that IL-18 T cells showed increased persistence compared to T cells expressing IL-12 under the control of the NFAT promoter [35]. The study also showed that IL-18-secreting T cells upregulated key costimulatory molecules and were able to prolong the survival of tumour-bearing mice.

Preclinical studies using tumour-specific human T cells engineered to express IL-15 in a glioblastoma model showed improvement in proliferation, persistence and cytokine production [36], while IL-21 engineering of human T cells showed enhanced persistence in a xenogeneic B-cell lymphoma model. [37]. It will be important to determine to what extent these cytokines enhance cancer immunity by improving the function of CD4+ and CD8+ T cells, respectively.

## 5. Costimulatory Domains and Blocking Inhibitory Signals

TCR engineered cells may have an attenuated response to tumour target cells due to insufficient costimulation. In addition, inhibitory receptors on the T-cell surface such as PD-1, LAG-3 and TIM-3 may bind to ligands that are expressed by tumour cells and actively impair T-cell responses [38,39,40]. There have been a number of studies using CRISPR–Cas9 to knock out inhibitory receptors such as PD-1, LAG-3 and TIM-3 in cancer-specific T cells, leading to enhanced anti-tumour activity [41,42,43].

An engineering technique with promise in preclinical studies is to convert T-cell inhibitory signals into stimulatory signals. T cell immunoreceptor with Ig and ITIM domains (TIGIT) is an inhibitory receptor that, when binding CD155 impairs T-cell activation. Using a xenograft model of human melanoma, it was shown that tumour-specific T cells expressing a hybrid molecule containing the TIGIT extracellular domain fused to the CD28 intracellular domain displayed enhanced production of IFN-γ and TNF-α and improved tumour control [44]. Similarly, the PD1 extracellular domain has been fused to the CD28 intracellular region and cotransduced with a cancer-specific TCR into human T cells. These engineered T cells exhibited enhanced cytokine secretion when cultured with PDL1-positive tumour cells [45]. Interestingly, CD4+ T cells engineered with the PD1/CD28 fusion construct provided enhanced T-cell help to improve the anti-tumour activity of CD8+ T cells in a pancreatic cancer and non-Hodgkin lymphoma model [46].

CD4+ T cells that are chronically stimulated by antigen exhibit an exhausted phenotype, with expression of many of the same inhibitory receptors also seen in exhausted CD8+ T cells [47]. However, a striking difference is that exhausted CD4+ T cells express much higher levels of CTLA-4 than their CD8+ counterparts. Recent experiments in murine cancer models showed that CTLA-4 blockade enhanced tumour immunity by improving the helper function of CD4+ T cells [48].

## 6. Enhancing Persistence and Memory Formation of Engineered T Cells

One of the barriers to lasting efficacy of genetically modified T-cell therapies is the lack of persistence of transferred cells in patients. Ineffective T-cell memory may be one of the factors leading to disease relapse. Hence, engineering approaches that to improve T-cell persistence and memory formation may prolong effective control of cancer progression.

Genetic engineering has been used to overexpress the chemokine receptor CXCR4 in tumour-specific CD8+ T cells, which promoted homing to bone marrow niches and preferential differentiation into memory T cells [49]. In a lymphoma model, the CXCR4-transduced T cells showed enhanced tumour protection compared to control T cells. [49]. Transient downregulation of mTORC1 signalling provides another strategy to enhance the formation of memory T cells. Using a genetic approach to transiently downregulate mTORC1 activity in engineered T cells demonstrated that this resulted in the enhanced formation of tumour-specific memory cells [50]. Alizadeh et al. showed that IL-15-engineered T cells displayed reduced mTORC1 activity that was associated with an increase in the frequency of stem-cell memory CD4+ and CD8+ T cells [51]. After adoptive transfer of the IL-15-engineered T cells, the reduced mTORC1 activity impaired the expression of exhaustion markers and prevented high level of CTLA4 expression in CD4+ T cells. Together, these observations suggest that strategies to reduce mTORC1 expression can improve memory formation by CD4+ and CD8+ T cells and prevent exhaustion in both subsets.

## 7. Enhancing Function by Altering T-Cell Metabolism

T-cell activation is associated with a metabolic switch from oxidative phophorylation to glycolysis [52]. This enables T cells to meet the increased energetic demands required for rapid proliferation and the development of effector functions. It is known that tumour microenvironments are hostile to both endogenous and adoptively transferred T cells due to the high metabolic activity of tumour cells, the depletion of glucose and glutamine, and the lack of key amino acids such as arginine and tryptophan, which are specifically depleted due to production of inhibitory enzymes such as arginase by the tumour cells. Gene-editing technologies provide an opportunity to improve T-cell metabolism in this challenging tumour microenvironment.

PPAR-γ co-activator 1α (PCG1α) can be overexpressed in T cells to allow mitochondrial function to be maintained, consequently leading to an increased cytokine production in T cells and enhanced anti-tumour activity [53]. Knocking out the gene encoding Acetyl-Coenzyme A acetyltransferase 1 (ACAT1), which is an enzyme involved in the esterification of cholesterol, was shown to improve the proliferation and effector function of cancer-reactive T cells. The improved anticancer activity of T cells was due to ACAT1 knockout resulting in increased membrane cholesterol, which improved TCR clustering and signal transduction [54].

Although, as discussed above, transient mTORC1 downmodulation can improve T-cell memory, genetic engineering to upregulate the activity of mTORC1 has been shown to improve the effector function of tumour-specific T cells. Ras homolog enriched in brain (RHEB) can be overexpressed in engineered T cells to increase mTORC1 signalling, which drives a metabolic switch to aerobic glycolysis and resulted in increased proliferation of effector T cells in vivo. It was demonstrated that RHEB-transduced T cells effectively accumulated in tumours, provided improved protection and reduced the emergence of immuno-edited tumour escape variants [50]. In this experimental model, the enhanced effector function of adoptively transferred T cells was associated with improved tumour control, although the memory formation of the RHEB-transduced T cells was impaired.

Signalling via mTORC1 also plays a role in the metabolic reprogramming of CD4+ T cells and their differentiation-cytokine-producing T-cell subsets. mTORC1 signalling promotes Th1 and Th17 differentiation, whilst mTOR inhibition promotes differentiation towards T regs [55]. CD4+ T cells that are deficient in RHEB are unable to secrete IFNγ, which prompted a study by the Powell lab investigating the role of the Th1 master transcription factor T box expressed in T cells (T-bet). They demonstrated that mTORC1 phosphorylates T-bet, and that when mTORC1 is inhibited, this suppresses T-bet-dependent IFNγ production [56]. In addition, the downstream target of mTORC1, Hypoxia inducible factor-1α (Hif-1 α), plays a role in limiting the effector function of CD4+ effector T cells, particularly in the case of Th1 cells [57].

Future engineering strategies will need to consider approaches that can sustain the effector function of adoptively transferred T cells in the tumour microenvironment, while also promoting the development of T-cell memory. This could be achieved by adoptive transfer of two distinct T-cell populations—one population engineered to increase mTOC1, which promotes T-cell differentiation and effector function, mixed with a population engineered to transiently inhibit mTOC1, which improves T-cell memory formation.

## 8. Conclusions and Future Directions

Over the last decade, the field of T-cell engineering has shown remarkable growth and an increasing sophistication in the strategies used to generate T-cell specificity and augment function. Techniques that aim to equip engineered T cells with a survival advantage in hostile tumour microenvironments have shown great promise, including those that work through augmentation of key effector cytokines and the use of molecular switches to improve the metabolic fitness of adoptively transferred T cells. In addition, gene-editing tools such as CRISPR–Cas9 provide an opportunity to selectively delete genes encoding proteins that negatively regulate tumour-protective T-cell effector function. The engineering platforms provide an opportunity to produce cancer-specific CD4+ T cells capable of recognising tumour cells via MHC class-I-restricted TCRs and contribute to protection by directly killing cancer cells and/or by improving the effector function and memory formation of CD8+ T cells.
